# Genetic Diversity and Population Structure of an Endemic Fish, *Nemacheilus subfusca*, in the Lower Yarlung Zangbo River Basin

**DOI:** 10.3390/ijms27177650

**Published:** 2026-08-26

**Authors:** Qi Su, Mantang Xiong, Yang Yang, Feng Chen, Sijin Jiang, Jianfang Liu, Zhiming Zhang

**Affiliations:** 1 Key Laboratory of Ecological Impacts of Hydraulic Projects and Restoration of Aquatic Ecosystem, Ministry of Water Resources, Hubei Engineering Research Center of Hydroecology Protection and Restoration, Institute of Hydroecology, Ministry of Water Resources and Chinese Academy of Sciences, Wuhan 430079, China; suqi@nwafu.edu.cn (Q.S.); xiongmantang@mail.ihe.ac.cn (M.X.); yyy13437137352@163.com (Y.Y.); chenfeng@mail.ihe.ac.cn (F.C.); jiangsijin001@163.com (S.J.); 18147048550@163.com (J.L.); 2 Innovation Team of the Changjiang Water Resources Commission for River and Lake Ecosystem Restoration Key Technology, Wuhan 430079, China

**Keywords:** *Nemacheilus subfusca*, mitochondrial gene, genetic diversity, geographical population, genetic structure

## Abstract

*Nemacheilus subfusca* is an endemic fish species in the lower Yarlung Zangbo River. Its habitats are potentially threatened by geological disturbances and anthropogenic activities, but information on population status remains limited. However, existing research has been largely confined to basic biology, with limited information on its population genetic structure and connectivity. Consequently, targeted investigations are urgently needed to provide a scientific basis for the conservation of this species. In this study, genetic diversity and population structure were analyzed in 50 individuals from two geographic populations (Motuo (MT) and Chayu (CY)) in the lower Yarlung Zangbo River, using mitochondrial *cytb* and *D-loop* markers. The populations exhibited high haplotype diversity but relatively low nucleotide diversity (*cytb*: *H_d_* = 0.870, *P_i_* = 0.00281; *D-loop*: *H_d_* = 0.931, *P_i_* = 0.01655). Notably, the MT population exhibited lower diversity levels compared to the CY population (*cytb*: *H_d_* = 0.757, *P_i_* = 0.00205; *D-loop*: *H_d_* = 0.633, *P_i_* = 0.00153). Substantial genetic differentiation was observed between the two populations (*cytb*: *F*_ST_ = 0.322; *D-loop*: *F*_ST_ = 0.733); The observed mitochondrial divergence did not provide clear evidence of species-level differentiation (*cytb* = 0.00331; *D-loop* = 0.02492). The ML phylogenetic tree and haplotype network revealed geographically associated clustering of the two populations, indicating geographic structuring of mitochondrial haplotypes. Furthermore, demographic analyses were consistent with a possible historical population expansion. In conclusion, The mitochondrial data revealed high haplotype diversity but relatively low nucleotide diversity, together with substantial genetic differentiation between the two sampled populations, forming geographically associated clusters. This study elucidates the genetic background of this species’ germplasm resources, providing baseline genetic information for future conservation and management of this species.

## 1. Introduction

The Yarlung Zangbo River basin harbors a unique and diverse fish fauna, predominantly characterized by the subfamily Schizothoracinae [1]. Previous population genetic studies in this basin have largely focused on these large-bodied, economically significant species. For instance, recent mitochondrial marker-based research on *Schizothorax curvilabiatus* has revealed significant genetic differentiation among populations across distinct geographic regions, highlighting a complex evolutionary history driven by the tectonic uplift of the Tibetan Plateau [2]. However, despite considerable advances in understanding these dominant schizothoracine species, the genetic patterns of small benthic fishes inhabiting tributaries remain poorly explored.

*Nemacheilus subfusca* (family Nemacheilidae, order Cypriniformes, is among the few nemacheilid fishes reported from the downstream tributaries of the Yarlung Zangbo River [3]. While the Tibetan region hosts 13 species of the genus *Triplophysa* and one species of *Aborichthys* [4,5,6], the two endemic species in the lower Yarlung Zangbo River—*Aborichthys kempi* and *N. subfusca*—are particularly understudied. *A. kempi* has only been reported in historical literature, with no recent records from resource surveys, and existing research is primarily conducted by Indian scholars [7,8,9]. Unlike the widely distributed Schizothoracines, *N. subfusca* has a highly restricted distribution, typically inhabiting crevices among gravel substrates or deep pools in sandy streams in tributary reaches [10,11]. Field observations conducted between 2022 and 2025 indicate that some habitats occupied by *N. subfuscus* are affected by anthropogenic activities and geological disturbances, highlighting potential conservation concerns. Furthermore, the absence of genetic data for this species represents a significant gap in our understanding of the regional fish fauna. Therefore, assessing the genetic diversity and population structure of *N. subfusca* is not only crucial for its targeted conservation, but also provides a more comprehensive perspective on the biogeographical processes shaping the fish assemblages of the Yarlung Zangbo River.

Genetic diversity is fundamental to a species’ adaptive capacity to environmental changes and serves as the cornerstone for formulating effective conservation strategies [12,13]. In fish population genetics, mitochondrial DNA (mtDNA) has been extensively utilized due to its maternal inheritance, lack of recombination, and moderate evolutionary rate [14,15]. To comprehensively capture the evolutionary history of this species, this study employed multiple molecular markers with varying evolutionary rates. The cytochrome b (*cytb*) gene, characterized by a relatively stable evolutionary rate, is highly effective for elucidating phylogenetic relationships and medium- to long-term evolutionary dynamics [16,17]. Conversely, the control region (*D-loop*), a highly variable non-coding mitochondrial region, provides high-resolution polymorphism data suitable for resolving recent population expansion events and fine-scale genetic structure [18,19,20,21].

Although genomic approaches such as RAD-seq can provide higher-resolution estimates of population structure, their application may be more technically demanding and resource-intensive in non-model species lacking genomic resources. Given the limited genomic resources available for this non-model species, we used two mitochondrial markers as a cost-effective approach for obtaining baseline information on maternal genetic diversity and population differentiation. Thus, the combined use of *cytb* and *D-loop* markers establishes a robust baseline for population genetics in the Yarlung Zangbo River, providing complementary information on mitochondrial variation and population history.

The levels of population genetic diversity, patterns of genetic differentiation among populations, and historical demographic processes within the natural distribution range of *N. subfusca* have yet to be systematically elucidated. We hypothesize that the complex topography and historical geological barriers in the Yarlung Zangbo basin have restricted gene flow, resulting in substantial mitochondrial differentiation and geographically structured genetic variation. We further considered whether recent anthropogenic disturbances could contribute to the observed population differentiation. To test these hypotheses, we analyzed 50 individuals sampled from the MT and CY sections. By jointly applying dual *cytb* and *D-loop* markers, we conducted a comprehensive analysis of genetic diversity and population structure. The findings provide baseline mitochondrial genetic information that may contribute to future conservation and management of *N. subfuscus* and help guide further population-genetic studies using nuclear markers.

## 2. Results

### 2.1. Sequence Characteristics of Mitochondrial Genes

Following calibration and comparison of the sequencing results, 1046 bp of *cytb* and 883 bp of *D-loop* sequences were selected for subsequent analysis. The mitochondrial sequences showed an A + T-rich nucleotide composition of the two populations of *N. subfusca*, with the A + T content considerably exceeding the G + C content (Table S1). This pattern is consistent with the A + T-rich nucleotide composition commonly reported for fish mitochondrial genomes. Additionally, the *cytb* sequence showed 24 polymorphic sites, comprising 12 parsimony informative sites and 12 singleton variable sites. The D-loop sequence contained 87 polymorphic sites, including 46 parsimony informative sites and 41 singleton variable sites.

### 2.2. Analysis of Population Genetic Diversity

The genetic diversity parameters of two populations of *N. subfusca* were analyzed using DnaSP 6.0. Among the 50 samples, a total of 15 *cytb* haplotypes were identified. For *cytb*, haplotype diversity was 0.757 in MT and 0.766 in CY, with an overall value of 0.870. For *cytb* the average nucleotide diversity index (*P_i_*) was 0.00205 in MT and 0.00245 in CY, with an overall value of 0.00281. For *cytb*, the average number of nucleotide differences (*K*) was 2.067 in MT and 2.468 in CY, with an overall value of 2.835. A total of 27 *D-loop* haplotypes was identified among the 50 individuals, including four haplotypes in the MT population (*n* = 21) and 23 haplotypes in the CY population (*n* = 29). The overall haplotype diversity (*H_d_*), nucleotide diversity (*P_i_*), and the average number of nucleotide differences (*K*) were 0.931, 0.01655, and 14.087, respectively. The D-loop region displayed higher absolute *H_d_*, *P_i_* and *K* values compared to *cytb*. This result is consistent with the generally faster evolutionary rate of the non-coding control region relative to protein-coding mitochondrial genes. The higher diversity observed in the *D-loop* is consistent with its generally higher evolutionary rate compared with the protein-coding *cytb* gene.

A comparative analysis based on single-gene data revealed that the haplotype diversity (*H_d_*), nucleotide diversity (*P_i_*), and average number of nucleotide differences (*K*) were consistently higher in the CY population than in the MT population.

### 2.3. Analysis of Population Genetic Structure

Integrating the results calculated using the DnaSP 6.0, statistical analyses of the inter-population genetic differentiation index, genetic distance, and gene flow yielded values of 0.32214, 0.00331, and 0.53 for the mitochondrial *cytb* gene, respectively. In contrast, the corresponding values for the D-loop region were 0.73316, 0.02492, and 0.09. Overall, both genes exhibited a consistent trend between the two populations, characterized by a high mitochondrial genetic differentiation, low *N*_m_ estimates, and relatively low genetic distances. However, the *D-loop* region yielded higher values for the differentiation index and genetic distance, along with a lower gene flow value compared to *cytb*, indicating a more pronounced level of inter-population differentiation.

The results of the AMOVA are presented in Table 1. The proportion of genetic variation within populations was 68.16% for *cytb* and 29.19% for *D-loop*, while the variation among populations was 31.84% for *cytb* and 70.81% for *D-loop*. These data indicate that the primary sources of genetic variation differ between the two genetic markers. For *cytb*, most genetic variation occurred within populations (68.16%), whereas for the *D-loop*, most variation occurred among populations (70.81%). Furthermore, the analysis of the genetic differentiation index indicated substantial genetic differentiation between the two sampled populations (*cytb*, *F*_ST_ = 0.31841; *D-loop*, *F*_ST_ = 0.70812; *p* < 0.05) (Table S2 and Table S3).

### 2.4. Haplotype Distribution and Phylogenetic Analysis

**Figure 1:**
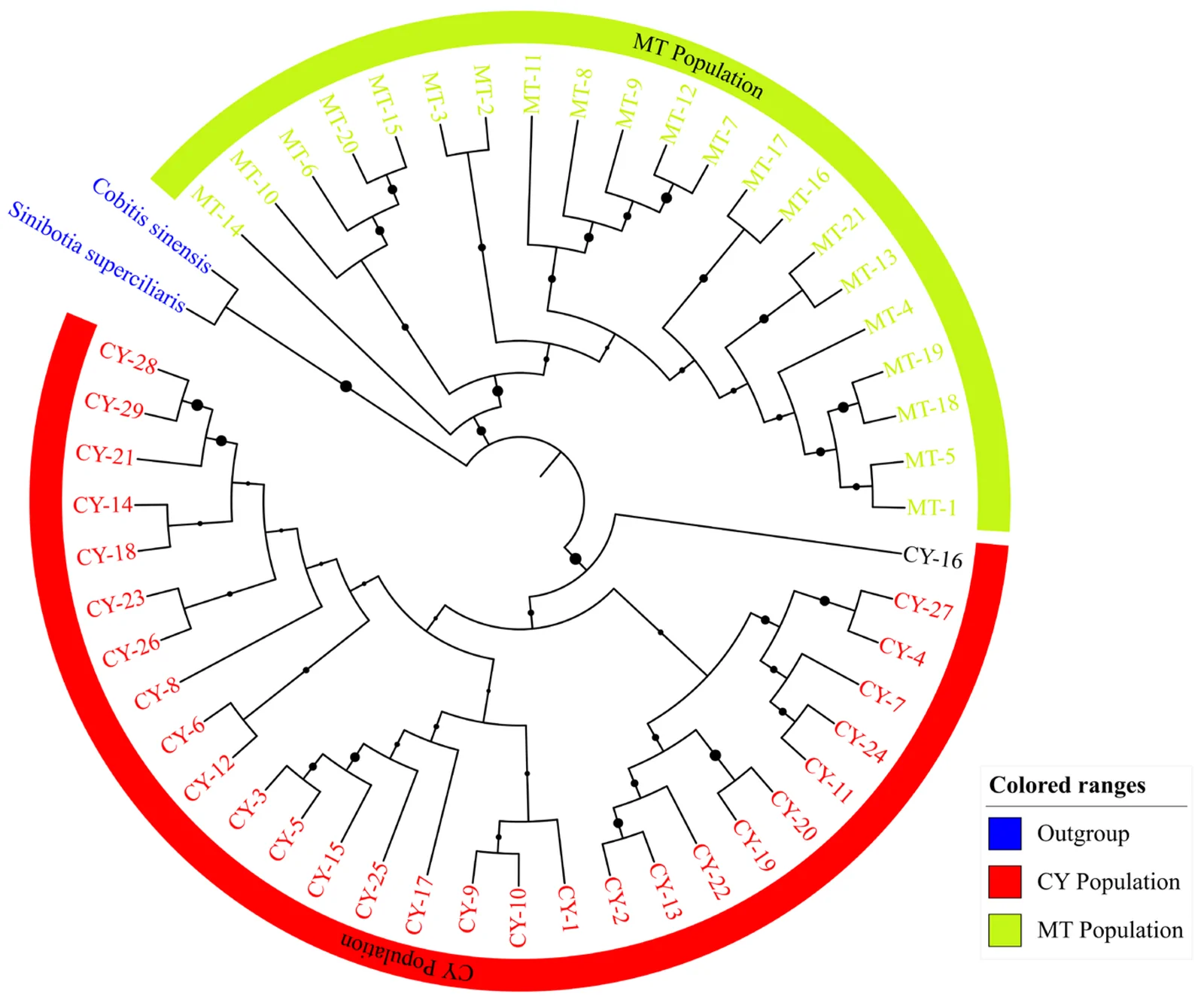
NJ phylogenetic trees based on cytb and D-loop sequences.

**Figure 2:**
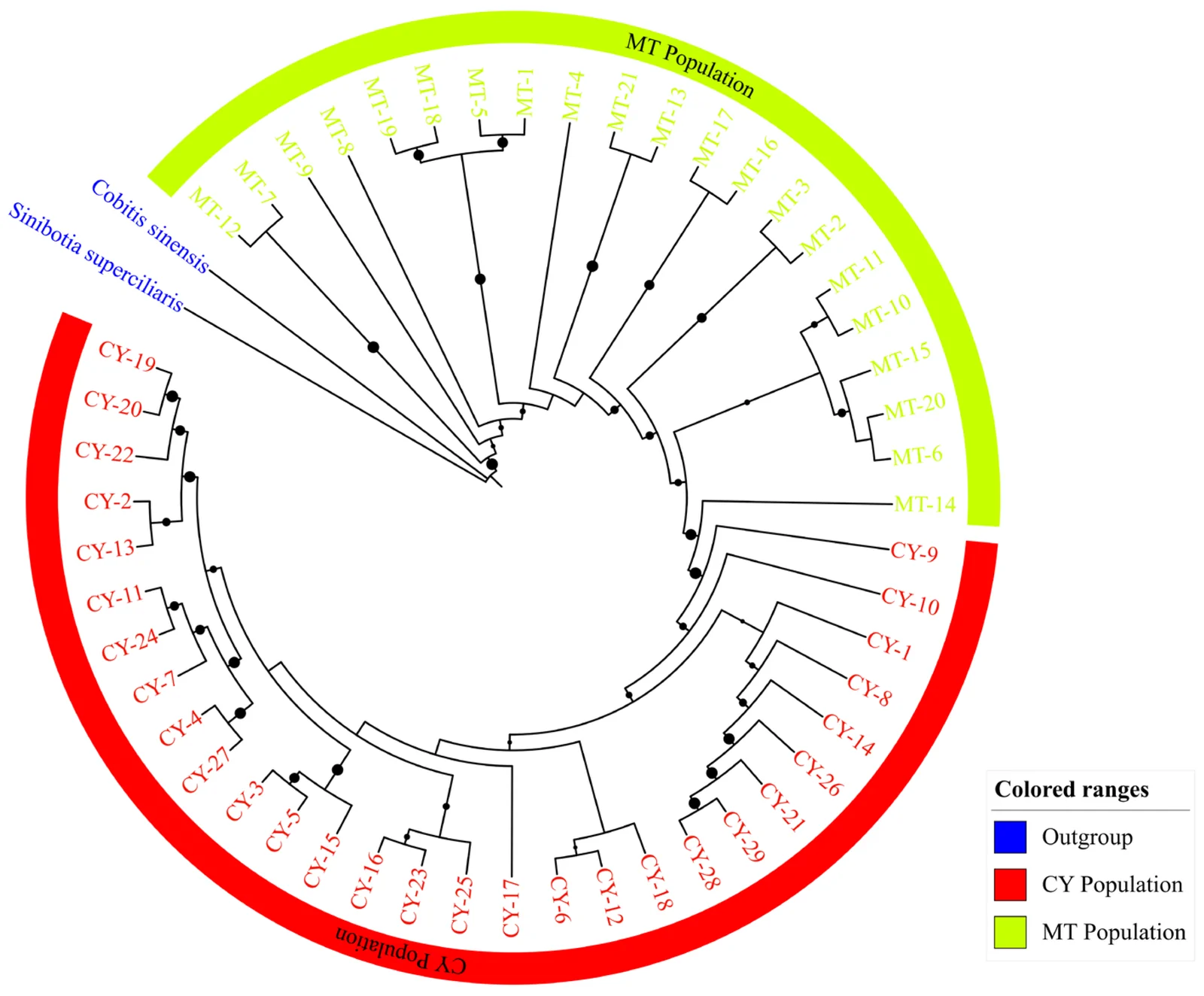
ML phylogenetic trees based on cytb and D-loop sequences. Note: The red represents the CY population (n = 29), Chartreuse represents the MT population (n = 21), blue represents the outgroup: *Sinibotia superciliaris* and *Cobitis sinensis*. Black circle size indicates bootstrap values (range: 4–100). Evolutionary model: TPM2 + F + R2; bootstrap replication number = 1000.

The Neighbor-Joining (NJ) phylogenetic tree and maximum likelihood (ML) tree of *N. subfusca*, constructed using two mitochondrial genes with *Sinibotia superciliaris* and *Cobitis sinensis* as outgroups, haplotypes from the two populations showed geographically associated clustering in both phylogenetic analyses [21,22], indicating substantial genetic differentiation between the two sampled populations (Figure 1 and Figure 2).

**Figure 3:**
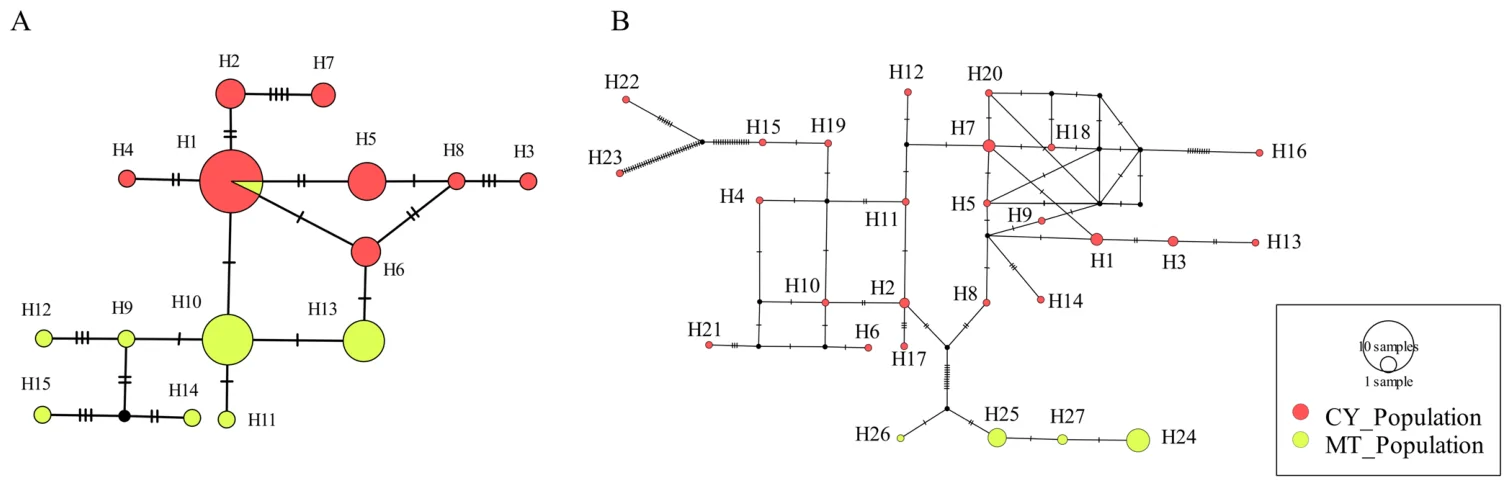
Median-joining network of *cytb* (**A**) and *D-loop* (**B**) haplotypes from two populations of *N. subfusca*.

To further characterize the relationships among haplotypes, median-joining networks were constructed based on the two mitochondrial genes (Figure 3). The analysis identified 15 haplotypes from the *cytb* gene and 27 from the *D-loop* region, exhibiting a distribution trend consistent with the NJ phylogenetic tree (Figure 1) and the ML phylogenetic tree (Figure 2). Notably, the *cytb* network revealed one shared haplotype between the MT and CY populations, whereas no shared haplotypes were observed between the two populations in the *D-loop* network. This result aligns with the aforementioned analyses, where the *cytb* gene exhibited lower values for the genetic differentiation index (*F*_ST_), genetic distance, and gene flow (*N_m_*) compared to the *D-loop* region.

### 2.5. Historical Demographic Analysis

The results of Tajima’s *D* and Fu’s *Fs* neutrality tests are presented in Table 2. For the *cytb* sequences, both Tajima’s *D* and Fu’s *Fs* values were negative in the two populations, with the *D* value being statistically significant only in the MT population (*p* < 0.05). In the overall analysis, the *D* and *Fs* values were −1.53316 (*p* < 0.05) and −4.47814 (*p* < 0.05), respectively. The analysis based on *D-loop* sequences showed that only the CY population exhibited significantly negative *D* and *Fs* values (*p* < 0.05). In the MT population, the *D* value was negative while the *Fs* value was positive, with neither being statistically significant. The overall analysis of the *D-loop* region yielded *D* and *Fs* values of −0.97214 (*p* > 0.05) and −2.59676 (*p* > 0.05), respectively.

**Figure 4:**
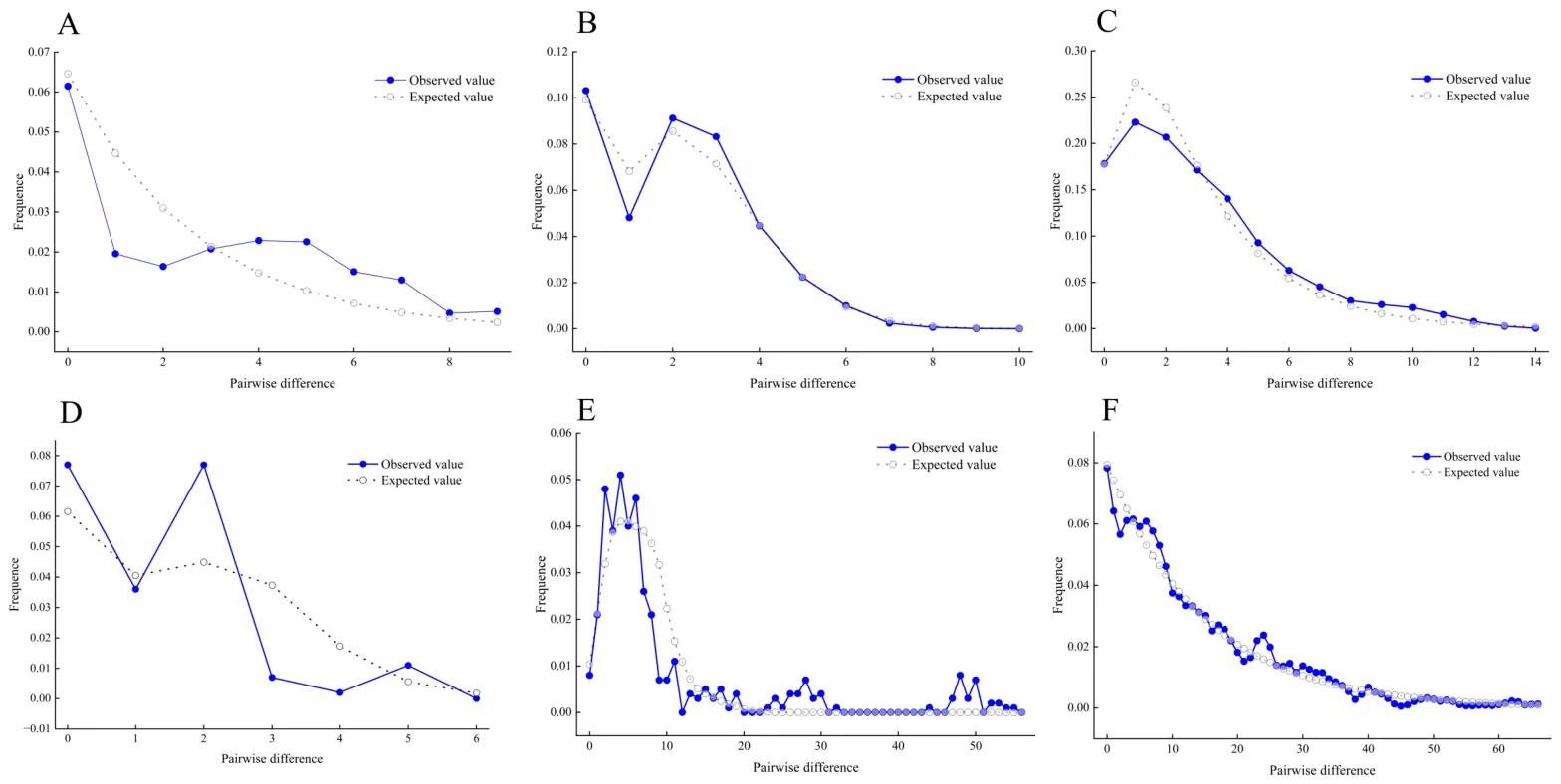
Mismatch distribution of *N. subfusca* based on mtDNA cytb gene and D-loop region sequences. (**A**) MT, (**B**) CY, (**C**) Total (*cytb*); (**D**) MT, (**E**) CY, (**F**) Total (*D-loop*).

Furthermore, the nucleotide mismatch distribution curves were multimodal in all analyses except for the overall *cytb* analysis, which showed an unimodal distribution (Figure 4A,B,D,E), The multimodal distributions observed for most datasets did not provide clear support for a simple sudden-expansion model. The sudden-expansion model was not rejected for the overall *cytb* dataset (*D* = −1.53316, *p* = 0.024; *Fs* = −4.47814, *p* = 0.039) (Table 3), and the overall nucleotide mismatch plot exhibited a unimodal pattern (Figure 4C), indicating that the mitochondrial data are consistent with a possible historical expansion, although additional nuclear markers are required to confirm demographic history. Although the Tajima’s *D* and Fu’s *Fs* values for the CY population based on the *D-loop* region were significantly negative, the mismatch distribution plot was multimodal (Figure 4F). Although the significantly negative neutrality statistics for the CY population are consistent with demographic expansion, the multimodal mismatch distribution does not provide unambiguous support for a simple sudden-expansion model.

## 3. Discussion

### 3.1. Genetic Diversity of Different Geographical Populations of N. subfusca

The mitochondrial data revealed high haplotype diversity but relatively low nucleotide diversity in both sampled populations of *N. subfusca*. Both mitochondrial markers exhibited an A + T-rich nucleotide composition, consistent with patterns commonly reported for fish mitochondrial DNA. This aligns with the nucleotide distribution patterns observed in the mitochondrial genomes of most fish species [22,23]. The assessment of genetic diversity revealed an overall pattern of high haplotype diversity (*H_d_*) and low nucleotide diversity (*P_i_*) in *N. subfusca*. This pattern is consistent with, but does not conclusively demonstrate, recent demographic expansion [24,25].

Notably, both *H_d_* and *P_i_* values in the *D-loop* region were higher than those in the *cytb* gene. The higher diversity observed in the *D-loop* is consistent with its generally higher evolutionary rate and greater polymorphism compared with protein-coding mitochondrial genes, which is consistent with the characteristics of non-coding regions subjected to weaker selective pressure and higher mutation rates, as well as conclusions from most fish mitochondrial studies [26,27]. In the inter-population comparison, the CY population exhibited relatively higher genetic diversity, whereas the MT population showed lower diversity. In plateau river systems, differences in altitude, climate, habitat structure, or other environmental factors could potentially contribute to the observed population differentiation; however, these factors were not evaluated in the present study [28,29]. Notably, previous research suggests that genetic diversity often declines in marginal distribution areas [2], offering a potential framework for understanding the population differentiation of *N. subfusca*. Despite these insights, the specific mechanisms governing the genetic differentiation patterns observed in this study warrant further elucidation.

### 3.2. Genetic Structure and Genetic Differentiation of Different Geographical Populations of N. subfusca

The genetic structure and differentiation among fish populations are comprehensively assessed using parameters such as genetic distance, gene flow, and the fixation index (*F*_ST_) [30]. In this study, both genes exhibited a consistent trend: the two populations showed high genetic differentiation indices and low gene flow, indicating substantial mitochondrial differentiation between the sampled populations. Although there is no absolute threshold, previous studies have suggested that genetic distances typically increase with taxonomic rank. Genetic distance values should be interpreted cautiously because thresholds separating population- and species-level divergence are taxon-dependent [31]. Restricted gene exchange due to geographical isolation or distinct habitat preferences within the same water system is a major driver of population divergence, gradually leading to genetic differentiation patterns that align with the species’ distribution [32,33].

As a primary indicator of genetic differentiation, *F*_ST_ is used to evaluate the degree of divergence between populations based on established thresholds [34,35]. The mitochondrial *F*_ST_ values indicated substantial differentiation between the MT and CY populations. Based on the analysis of the two genes, the *F*_ST_ values between the two populations were 0.32214 (*cytb*) and 0.73316 (*D-loop*), respectively, indicating a high level of genetic differentiation. Additionally, gene flow (*N_m_*) serves as a crucial metric for assessing population structure. In this study, the *N_m_* values between the MT and CY populations were 0.53 and 0.09, respectively. The low *N_m_* estimates derived from mitochondrial FST are consistent with limited maternal connectivity between the sampled populations, although these estimates should not be interpreted as direct measures of contemporary migration [36].

Synthesizing the results of genetic distance, *F*_ST_, and gene flow estimates, the mitochondrial data collectively indicate strong genetic differentiation and limited maternal gene flow between the MT and CY populations. However, the Analysis of Molecular Variance (AMOVA) revealed contrasting patterns of genetic structure between the two markers: 68.16% of the genetic variation in the *cytb* gene was attributed to differences within populations, whereas the *D-loop* region exhibited a reversed pattern, with 70.81% of the variation occurring among populations. The contrasting AMOVA patterns between *cytb* and *D-loop* may reflect differences in mutation rate, sequence variability, stochastic lineage sorting, or sampling effects. Because only two mitochondrial loci were analyzed and no nuclear markers or environmental variables were included, the biological basis of this marker-specific discordance cannot be resolved in the present study.

### 3.3. Population Historical Dynamics of Different Geographical Populations of N. subfusca

In this study, neutrality tests and nucleotide mismatch distribution analyses based on two genetic markers were employed to infer historical demographic events. Significantly negative Tajima’s *D* and Fu’s *Fs* values indicate departures from the neutral equilibrium model and may be consistent with demographic expansion, although alternative explanations such as selection and population structure cannot be excluded [37]. When coupled with a unimodal mismatch distribution, these results provide additional support for a possible historical expansion [38].

Our results for the *cytb* gene—characterized by significantly negative neutrality tests and a unimodal mismatch distribution—are consistent with a possible historical expansion in the sampled populations. We acknowledge, however, that population structure, selective sweeps, or sampling biases could potentially influence these signals. In contrast, the *D-loop* region of the CY population presented a discordant pattern: the significant negative neutrality statistics for CY are consistent with demographic expansion, whereas the multimodal mismatch distribution does not provide unambiguous support for a simple sudden-expansion model. Therefore, the demographic history of the CY population remains uncertain.

The complex geological history of the Yarlung Zangbo River basin provides several plausible mechanisms that could have contributed to the observed mitochondrial structure. Historical river capture, tectonic activity, and Quaternary climatic fluctuations may have altered connectivity among tributaries and influenced population persistence. However, because the present study did not incorporate geological reconstructions, palaeoclimatic data, environmental variables, or demographic modelling, these mechanisms remain hypotheses that require independent testing.

In summary, the *cytb* results provided mitochondrial evidence consistent with a possible historical expansion in the sampled populations. Similarly, the distinct patterns observed in the hypervariable *D-loop* region reflect the complex evolutionary dynamics of local populations; however, these differences may also arise from its higher mutation rate, stochastic processes, or sampling effects, rather than strictly independent evolutionary histories.

## 4. Materials and Methods

### 4.1. Sample Collection and DNA Extraction

In this study, *N. subfusca* was identified based on morphological characteristics. The diagnostic features include an elongated, slightly compressed body and a short caudal peduncle that maintains an almost uniform depth towards the caudal fin. The mouth is inferior, featuring a single tooth-like projection in the middle of the upper jaw. The body is covered with small scales, with lateral line pores being sparse or absent on the posterior trunk. The sides of the body are marked with multiple brown crossbars that are uniform in width and evenly distributed. Additionally, the caudal fin is deeply emarginate with a dark brown crossbar at its base.

Sampling was conducted at two geographic locations: the MT sections of the Yarlung Zangbo River and the Chayu River. At each location, sampling sites were selected within the known distribution range of *N. subfusca*. To ensure the representativeness of the samples, individuals were randomly collected from the available habitats (including riffles and pools) within these sections using fish traps. We treated the samples from each river as distinct geographic populations (hereafter referred to as the MT population and the CY population).

**Figure 5:**
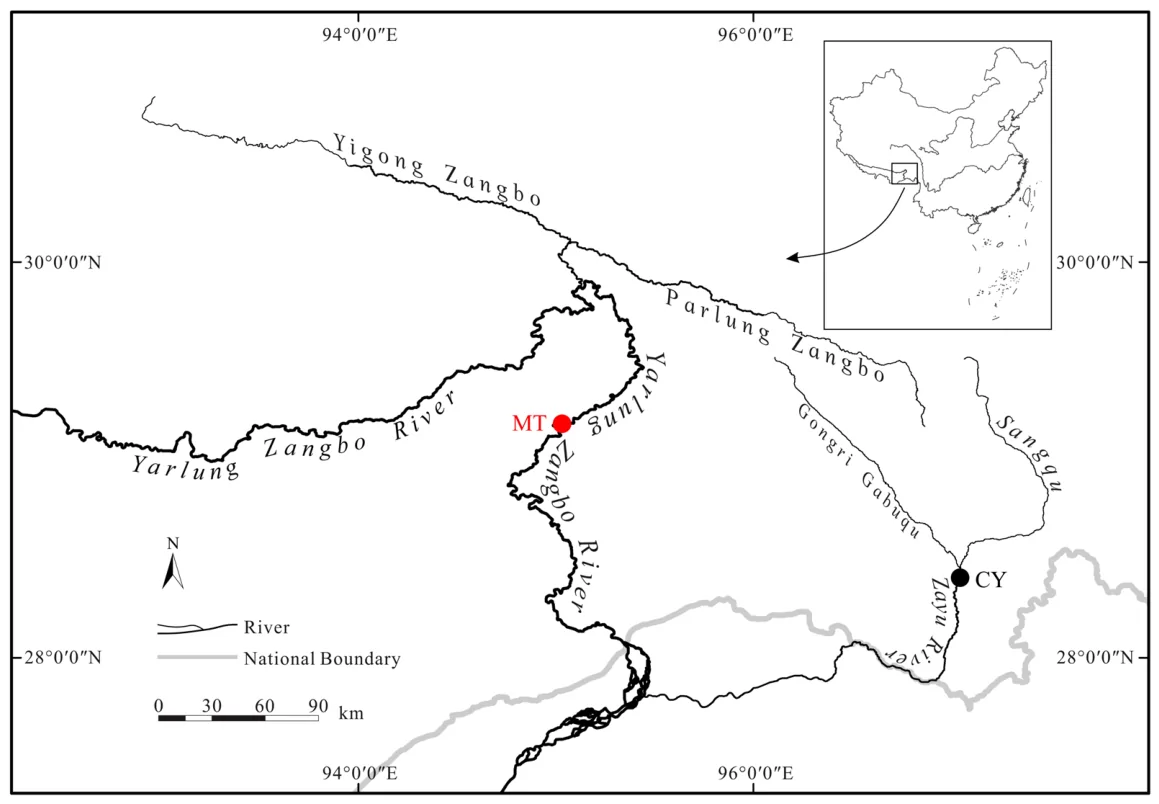
Sample sites of *N. subfusca.* Note: The red points represent the MT population; the black points represent the CY population.

**Figure 6:**
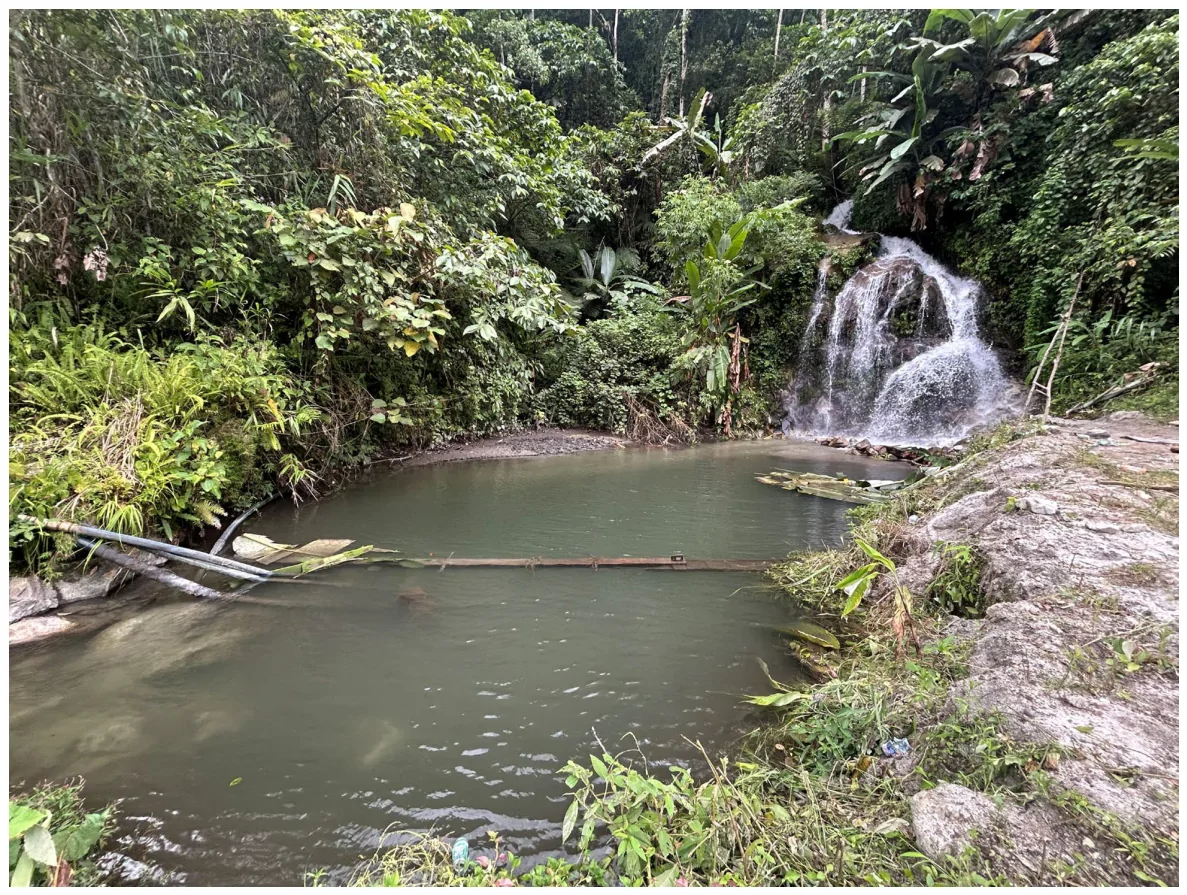
Habitat characteristics of the MT population of *N*. *subfusca*.

**Figure 7:**
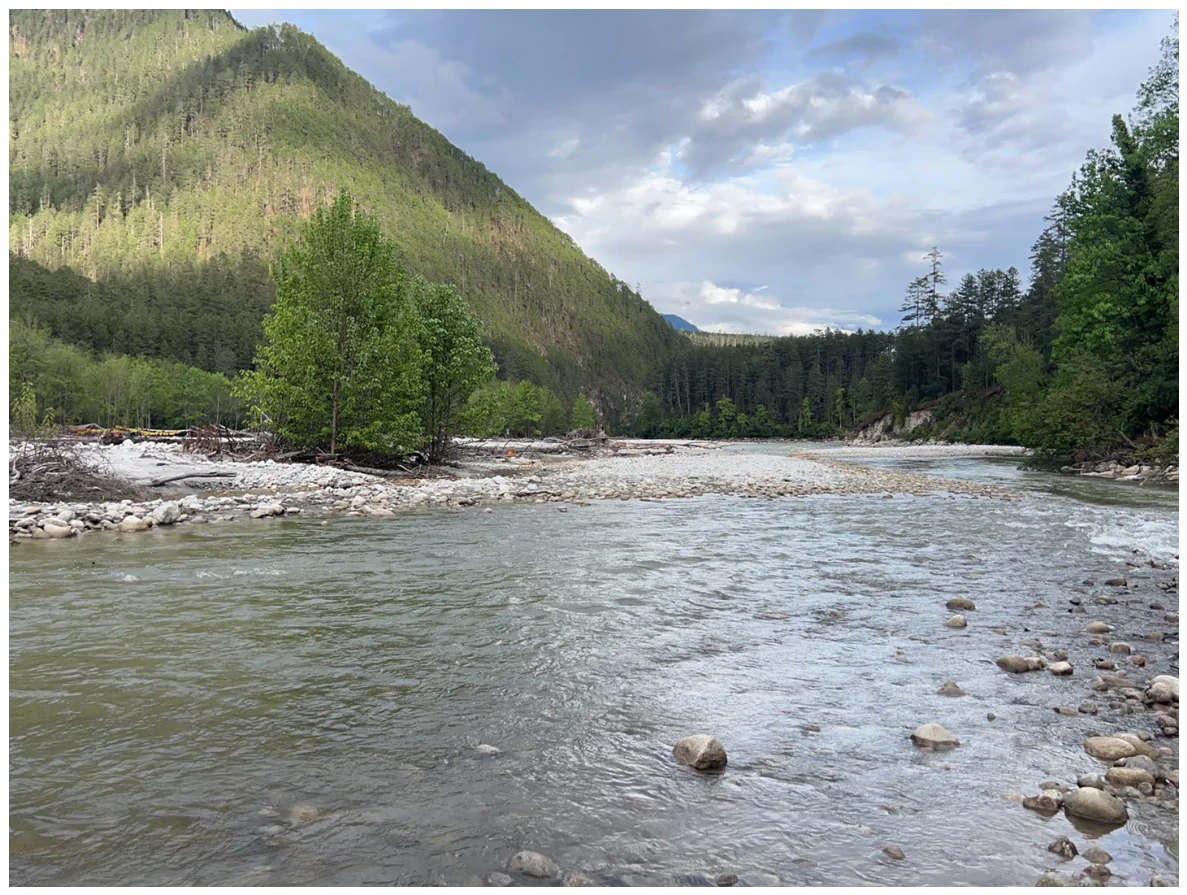
Habitat characteristics of the CY population of *N. subfusca*.

Following morphological identification, pectoral fin clips were collected for DNA extraction. The *N. subfusca* samples were obtained from the MT sections of the Yarlung Zangbo River (*n* = 21) and Chayu River (*n* = 29) in the lower reaches of the Yarlung Zangbo River (Table 3; Figure 5; detailed sampling information in Table 3 and Figure 5, Figure 6 and Figure 7). Fin clips were preserved in anhydrous ethanol (≥99.5%) and stored at −20 °C. Genomic DNA was extracted using the Biospin Genomic DNA Extraction Kit-BS7011 (Sangon Biotech, Shanghai, China).

To ensure the reliability of downstream applications, strict quality control (QC) was performed on the extracted DNA. DNA concentration and purity were sufficient for PCR amplification and downstream Sanger sequencing. An optical density (OD) A260/280 ratio between 1.8 and 2.0, and a minimum concentration threshold (e.g., >20 ng/μL) were used. DNA integrity was assessed by 1% agarose gel electrophoresis to exclude severely degraded samples. Samples yielding successful amplification of the target fragments were retained for subsequent analyses.

### 4.2. cytb and D-loop Region Sequence Primer Design and PCR Amplification

Primers were designed using Primer 6 software (Premier Biosoft, Palo Alto, CA, USA) based on the *cytb* and *D-loop* sequences (GeneBank Accession no. NC_031378.1) from NCBI. The design criteria were optimized as follows: expected amplicon length (*cytb*: 1100 bp; *D-loop*: 883 bp), melting temperature ranging from 58 °C to 62 °C, and GC content between 40% and 60%. To ensure specificity, the primer sequences were subjected to Web BLAST(NCBI) 2.16.0 analysis against the NCBI nucleotide database, confirming high specificity to the target gene, as detailed in Table 4. All primers were synthesized by Sangon Biotech, Shanghai, China.

The PCR reaction consisted of a total volume of 20 μL, including 10 μL of 2× Taq PCR Master Mix (with dye), 6.5 μL of ddH_2_O, 1 μL each of upstream and downstream primers (at a concentration of 10 μmol/L), and 1.5 μL of template DNA. The PCR cycling conditions were as follows: an initial denaturation at 95 °C for 4 min, followed by 35 cycles of denaturation at 95 °C for 30 s, annealing at 55 °C for 30 s, and extension at 72 °C for 40 s. A final extension was performed at 72 °C for 10 min, followed by a hold at 15 °C for 5 min. The PCR products were subsequently sent to Biomarker Technologies Co., Ltd (Beijing, China) for sequencing.

To ensure the reliability of the results, no-template controls (NTCs) containing nuclease-free water were included in each PCR run to detect potential contamination. All PCR amplifications were performed in triplicate (technical replicates) to ensure amplification success. The reaction yielding the most robust and specific product was selected for purification and sequencing. The PCR products were visualized on a 1.5% agarose gel, and the target bands were excised and purified using a Gel Extraction Kit (Sangon Biotech, Shanghai, China) to remove residual primers, dNTPs, and non-specific fragments before being sent for sequencing.

Sequencing was performed on an ABI 3730xl DNA Analyzer (Applied Biosystems, Carlsbad, CA, USA) by Biomarker Technologies Co., Ltd (Beijing, China). Raw reads were assembled and edited using (Bioedit 7.0.5). To ensure data accuracy, sequences with a Phred quality score below 20 were excluded from downstream analysis. For heterozygous sites, base calls were confirmed by bidirectional sequencing; any remaining ambiguous positions were coded using standard IUPAC ambiguity codes.

All nucleotide sequences obtained in this study have been deposited in the GenBank database. The GenBank accession numbers for the *D-loop* sequences range from PZ867331 to PZ867380, and those for the *cytb* sequences range from PZ867281 to PZ867330.

### 4.3. Data Analysis

Sequences were edited using BioEdit 7.0.5 and verified against reference mtDNA (GenBank Accession no. NC_031378.1) in MEGA 7.0 [39]. Protein-coding *cytb* sequences were aligned using ClustalW with default parameters, while *D-loop* sequences were aligned using MAFFT 7.0 (E-INS-i algorithm) and refined with Gblocks v.0.91b (relaxed parameters) to remove positions with >50% gaps [40,41,42]. Indels were treated as phylogenetic characters using Simple Indel Coding in SeqState v.1.1 and concatenated with the nucleotide alignment, resulting in a final dataset of 50 sequences (883 bp) [43,44]. The best-fit evolutionary model (TPM2 + F + R2) was selected using ModelFinder Plus in IQ-TREE for Maximum Likelihood (ML) analysis (1000 ultrafast bootstrap replicates) [45]. Additionally, a Neighbor-Joining (NJ) tree was constructed in MEGA 7.0 using the Kimura 2-parameter (K2P) model with 1000 bootstrap replicates; bootstrap values >70% are displayed in Figure 1 [46]. Genetic diversity indices (*S*, *P_i_*, *H_d_*) were calculated using DnaSP 6.0, and a haplotype network was constructed using PopART 1.7 [47,48]. Inter-population genetic distances (K2P) were analyzed in MEGA 7.0, yielding average distances of 0.00331 (*cytb*) and 0.02492 (*D-loop*). Genetic structure was quantified via Analysis of Molecular Variance (AMOVA) in Arlequin 3.5 using the Tamura–Nei model (10,000 permutations), and *F*_ST_ was calculated using Wright’s Fixation Index. Gene flow (*N_m_*) was estimated as *N_m_* = 0.5(1/*F*_ST_ − 1), interpreted as a theoretical estimate of historical maternal gene flow [49]. Finally, demographic history was inferred using Tajima’s *D* and Fu’s *Fs* statistics in DnaSP, while mismatch distribution analysis was performed in Arlequin (1000 bootstrap replicates) to test for sudden population expansion [50,51].

## 5. Conclusions

In conclusion, mitochondrial DNA markers reveal that *N. subfusca* in the lower Yarlung Zangbo River exhibits high haplotype diversity but relatively low nucleotide diversity, suggesting the presence of multiple haplotypes with limited sequence divergence. Furthermore, the two sampled populations showed substantial mitochondrial genetic differentiation and geographically structured haplotype distributions. These findings provide baseline mitochondrial genetic information for future conservation and management of the species. While this study focused on mitochondrial variation in two sampled populations, future research incorporating nuclear genetic markers and broader geographic sampling would help refine our understanding of the species’ demographic history and population structure.

## Figures and Tables

**Table 1:** Analysis of Molecular Variance (AMOVA).

Gene	Source of Variation	Degree of Freedom	Sum of Squares	Variance Components	Percentage of Variation	Fixation Index
*cytb*	Among populations	1	14.242	0.53741	31.84	*F*_ST_ = 0.31841
	Within population	48	55.218	1.15038	68.16	
	Total	49	69.460	1.68779	100	
*D-loop*	Among populations	1	191.885	7.74601	70.81	*F*_ST_ = 0.70812
	Within population	48	153.255	3.19280	29.19	
	Total	49	345.140	10.93881	100	

**Table 2:** Neutrality test for two *N. subfusca* populations.

Gene	Neutral Test	MT	CY	Total
*cytb*	*D*	−1.69571 *	−0.63213	−1.53316 *
	*p*	0.025	0.297	0.024
	*Fs*	−1.95743	−0.64485	−4.47814 *
	*p*	0.115	0.387	0.039
*D-loop*	*D*	−0.18435	−1.81204 *	−0.97214
	*p*	0.472	0.009	0.125
	*Fs*	0.68523	−8.2888 **	−2.59676
	*p*	0.659	0.004	0.235

Note: * represents *p* < 0.05; ** represents *p* < 0.01.

**Table 3:** Sample collection information of *N. subfusca*.

Population	Longitude	Latitude	Sample Size	Collected Randomly	Collection Method	Adults/Juveniles	Sample Time	Abbreviation
MT Population	95.0898242	29.20034825	21	random sampling	fish traps	adults	2025.08	MT
CY Population	97.03937657	28.39655278	29	random sampling	fish traps	adults	2025.08	CY

**Table 4:** Mitochondrial gene primer sequence information.

Genes		Primer Sequences (From 5′ to 3′)	GenBank Accession No.	Size (bp)
*cytb*	Forward	GGCAAGCCTACGAAAAACCC	NC_031378.1	1100
	Reverse	TCCTGCTAGTGGCATCAAGA		
*D-Loop*	Forward	TCTCACCACTGGCTCCCAA	NC_031378.1	883
	Reverse	TTGGGCTATATTTTGGCGTGA		

## Data Availability

The original contributions presented in this study are included in the article/Supplementary Materials. Further inquiries can be directed to the corresponding author.

## Supplementary Materials

The following supporting information can be downloaded at https://www.mdpi.com/article/10.3390/ijms27177650/s1.
