# Supplementary material for: Genetic Diversity and Population Structure of an Endemic Fish, Nemacheilus subfusca, in the Lower Yarlung Zangbo River Basin
Source: Int J Mol Sci. 2026 Aug 26;27(17):7650. doi: 10.3390/ijms27177650 (PMC13566335; doi:10.3390/ijms27177650)
Supplement: Supplementary file 1 [file ijms-27-07650-s001.zip › ijms-4403603-supplementary.pdf]

Genetic Diversity and Population Structure of *Nemacheilus subfuscus* in the Lower Yarlung Zangbo River Basin

Supplementary materials

Table S1. Nucleotide composition of mtDNA cyt*b* and *D-loop* gene sequences of *N. subfuscus.*

Genes	Base	MT(Percentage)	CY(Percentage)	Total(Percentage)
Cyt *b*	A	28.4	28.4	28.4
	T	28.1	28.1	28.1
	G	15.1	15.0	15.0
	C	28.5	28.5	28.5
	A+T	56.5	56.5	56.5
D-loop	A	33.1	32.7	32.9
	T	33.9	33.9	33.8
	G	13.0	13.2	13.1
	C	20.0	20.3	20.1
	A+T	67.0	66.6	66.7

Table S2. F_{ST}-value calculation matrix forof mtDNA cyt*b* and *D-loop* gene sequences of *N. subfuscus.*

Gene	F_{ST}-value	MT population	CY population
cytb	MT population	0	
	CY population	0.31841	0
D-loop	MT population	0	
	CY population	0.70812	0

Table S3. *P-value calculation matrix for the mtDNA Cyt b and D-loop gene sequences of N. subfuscus*

Gene	P-value	MT population	CY population
cytb	MT population	*	
	CY population	0.0000+-0.0000	*
D-loop	MT population	*	
	CY population	0.0000+-0.0000	*
